# Acute Myeloid Leukaemia Drives Metabolic Changes in the Bone Marrow Niche

**DOI:** 10.3389/fonc.2022.924567

**Published:** 2022-06-29

**Authors:** Rebecca S. Maynard, Charlotte Hellmich, Kristian M. Bowles, Stuart A. Rushworth

**Affiliations:** ^1^Norwich Medical School, University of East Anglia, Norwich Research Park, Norwich, United Kingdom; ^2^Department of Haematology, Norfolk and Norwich University Hospitals NHS Trust, Norwich, United Kingdom

**Keywords:** acute myeloid leukaemia, bone marrow niche, metabolism, adipocytes, free fattty acids

## Abstract

Acute myeloid leukaemia (AML) is a highly proliferative cancer characterised by infiltration of immature haematopoietic cells in the bone marrow (BM). AML predominantly affects older people and outcomes, particularly in this difficult to treat population remain poor, in part due to inadequate response to therapy, and treatment toxicity. Normal haematopoiesis is supported by numerous support cells within the BM microenvironment or niche, including adipocytes, stromal cells and endothelial cells. In steady state haematopoiesis, haematopoietic stem cells (HSCs) primarily acquire ATP through glycolysis. However, during stress-responses HSCs rapidly transition to oxidative phosphorylation, enabled by mitochondrial plasticity. Historically it was thought that cancer cells preferentially used glycolysis for ATP production, however recently it has become evident that many cancers, including AML primarily use the TCA cycle and oxidative phosphorylation for rapid proliferation. AML cells hijack the stress-response pathways of their non-malignant counterparts, utilising mitochondrial changes to drive expansion. In addition, amino acids are also utilised by leukaemic stem cells to aid their metabolic output. Together, these processes allow AML cells to maximise their ATP production, using multiple metabolites and fuelling rapid cell turnover which is a hallmark of the disease. This review of AML derived changes in the BM niche, which enable enhanced metabolism, will consider the important pathways and discuss future challenges with a view to understanding how AML cells are able to hijack metabolic pathways and how we may elucidate new targets for potential therapies.

## Introduction

AML is characterised by the malignant transformation of haematopoietic cells, which reside in the BM and ‘take over’ the environment of healthy haematopoietic stem and progenitor cells (1). AML is the most common acute leukaemia in adults and its incidence increases with age. Although treatment options have improved in the last decades (2), the prognosis for patients with AML remains poor, with high rates of relapse in patients who achieve initial remission, and many older patients unable to receive the most intensive treatments available (3, 4). It is therefore imperative that we further our understanding of the mechanisms of the disease, to elucidate more targets for treatment and drive the development of more effective and less toxic treatments.

One area of study is the metabolic changes that occur in the leukaemic BM niche. The BM is a structurally complex organ of blood vessels and a heterogeneous population of haematopoietic cells as well numerous support cells, including mesenchymal stromal cells (MSC), adipocytes, endothelial cells and osteoblasts (5). This creates the tissue specific niche to support the production, differentiation and maintenance of haematopoietic stem and their progeny (6) both in homeostasis and when required in response to systemic stresses. Of note the HSC niche is a hypoxic environment due to atrial blood being relatively deoxygenated when reaches the BM (7). This hypoxic state benefits long-term HSC health as it reduces oxidative stress and promotes HSC quiescence (8) and despite these relatively hypoxic conditions HSC are able to produce sufficient ATP for homeostasis (9).

A key function of HSCs is their ability to rapidly respond to external stress such as bleeding or infection and to increase their energy production in order to support rapid HSC expansion and production of mature blood cells. To achieve this, HSCs can transition rapidly to oxidative phosphorylation (OXPHOS) by acquiring mitochondria from BM stromal cells and increasing the uptake of other metabolites such as free fatty acids (FFA) and amino acids (10–12). We and others have shown that leukaemic cells are able to hijack these metabolic pathways in order to drive leukaemogenesis in preference to normal haematopoiesis (11, 13–19). This allows leukaemic cells to increase their metabolic output and ATP production and thus their proliferative potential. AML blasts also interact with the supporting BM cell populations and induce molecular changes to transform the normal haematopoietic niche into a ‘leukaemic niche’, which favours leukaemic growth over normal haematopoiesis (20). Here we will review the metabolism of the BM niche and how this changes in AML to promote leukaemogenesis. First, we will discuss the more permissible niche during normal metabolism of HSCs in the steady state and in response to stress stimuli. We will then contrast this with the leukaemic niche and how AML utilises the physiological pathways to its advantage. Finally, we will highlight new potential therapeutic targets based on these metabolic abnormalities for treating patients with leukaemia.

## Bone Marrow Niche

Haematopoiesis occurs within the BM niche, a supportive microenvironment that helps to regulate maintenance of HSCs and progenitor populations as well as blood cell production (6). Blood cell turnover is high, with 500 billion cells being produced daily (21), this process therefore not only needs to be regulated but also requires a constant and reliable energy supply. The supportive components of the BM niche provide a stable environment for this, as well as protecting HSCs from over-stimulation (22) and driving the required changes in cell expansion and differentiation in response to both local and systemic stress.

**Figure 1:**
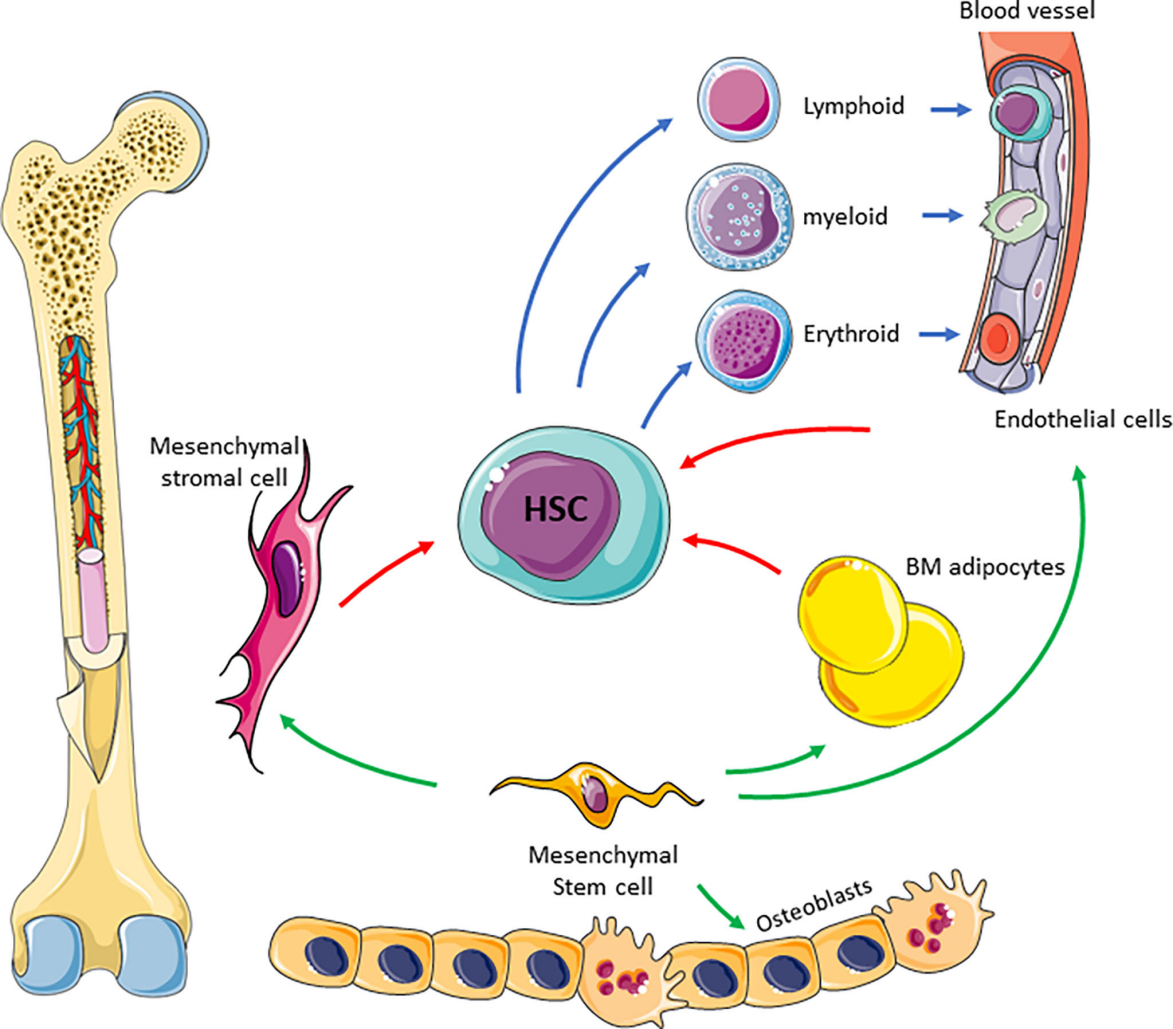
Schematic of BM niche. The complexity of the niche is highlighted showing many cell types and interaction between these cells to facilitate blood production. Mesenchymal stem cells differentiate into BM adipocytes, stromal cells, endothelial cells and osteoblasts (green arrow). Mesenchymal derived cells supports HSC differentiation (red arrows) in to blood progenitor cells (blue arrows).

The complexity of the supportive BM niche is illustrated in **Figure 1**. The BM niche comprises many cell types including mesynchymal stem cells which are multipotent and differentiate into adipocytes, endothelial cells, MSC, osteoblasts, myocytes and chrondrocytes (23). Most of these cells directly interact with HSCs through cytokines and chemokines to regulate their maintenance and differentiation (6). Adipocytes are derived from MSCs and store high levels of fat as triglycerides in lipid droplets (24). They therefore provide an important source of energy, as well as regulating energy metabolism of HSCs and other cells residing in the BM niche (25). Endothelial cells form the lining of blood vessels and thus form the interface between circulating blood and the BM niche (5). They regulate trafficking and homing of HSCs and progenitor cells and secrete a number of cytokines, including granulocyte colony stimulating factor (G-CSF), granulocyte-macrophage colony stimulating factor (GM-CSF) and interleukin 6 (26). Finally, the osteolinage cells, including osteoblasts and osteoclasts, reside within the BM niche and are responsible for bone formation and resorption, which occurs alongside haematopoieisis within an overlapping microenvironment (27, 28). Whilst osteoblasts are thought to only be indirectly involved in the regulation of haematopoiesis (6), osteoclasts are not only derived from monocyte precursors (28) but have also been shown to be vital for HSC homing (29). All of the individual components of the BM niche work together to co-ordinate haematopoieisis, regulate HSC quiescence and drive differentiation.

## Metabolism in the Bone Marrow Niche

Haematopoiesis is a dynamic process and cellular proliferation must respond to changes in the BM microenvironment as well as systemic changes and stress stimuli (5). Quiescent HSCs maintain a mitochondrial mass regulated by interplay between biogenesis and mitophagy (10) with low levels of reactive oxygen species (ROS) driving HSCs to favour anaerobic glycolysis. However, the metabolic conditions of HSCs change rapidly as they react to external systemic stressors such as infection or bleeding, amplifying blood production with minimal delay. In response to such stressors HSCs switch from glycolysis to OXPHOS to allow rapid HSC expansion and a several fold increase in production of mature haematopoietic cells (30) with an increase in ROS promoting HSC differentiation (10, 30). To support this switch to OXPHOS and supply the energy required, HSCs increase their mitochondrial mass initially *via* the transfer of mitochondria from BM stromal cells to HSCs *via* Connexin 43 Gap Junctions (10). Increased ROS levels have been shown to mediate PI3 kinase activation and subsequent gap junction formation (31). The influx of mitochondria from the environment supports the initial increase in energy demand and is only later in the response to stress that genes involved mitochondrial biogenesis are found to be upregulated (10). Thus, an increase in overall mitochondrial mass drives rapid HSC cycling and differentiation in response to pathogenic stress. Not all HSCs differentiate and a pool of HSCs remain metabolically quiescent and undifferentiated.

Furthermore, HSCs are able to utilise different metabolites within the BM niche to support their self-renewal and differentiation (32–35). BM adipocytes originate from mesenchymal stem cells (36) and with increasing age the proportion of BM mass occupied by adipose tissue gradually increases and can exceed 70% of the bone marrow compartment in older people (37, 38). The BM adipose tissue not only has a function in supplying energy but also as component of the endocrine system (39–41). The release of adipokines and inflammatory factors can alter the proliferative properties of the interacting haematopoietic cells (42). These factors form part of the signalling network that drives HSC differentiation and production of progenitor and mature haematopoietic cells during stress. At the same time lipolysis in BM adipose tissue provides a source of FFA for CPT1a mediated β-oxidation, which has been shown to play a role in regulating HSC asymmetric division and maintenance (32), and provide an additional source of energy to drive their expansion (43). A number of different proteins are involved in the transfer of FFA from the adipocytes, including CD206, CD36, fatty-acid-binding proteins (FABPs), and fatty-acid transport proteins (FATPs) (11, 44–46). In response to infection HSCs rely on the upregulation of the inducible fatty acid transporter, CD36, to facilitate the influx of FFA into HSCs. This promotes a further shift in the metabolic profile of the HSC from glycolysis at its steady state to β-oxidation in a stressed state (11). Together with the increase in OXPHOS this supports HSCs to maximise ATP production and facilitates rapid emergency haematopoiesis.

Amino acids are essential for HSC homeostasis and maintenance (34, 47). They are not only required for protein synthesis but also function as intermediate metabolites. They play a role in the interconnected metabolic pathways utilised by HSC, especially in response to stress and can directly enter the TCA cycle by generating acetyl-CoA (48). This can maintain the TCA cycle without the need for other substrates and thus amino acids can contribute in the same manner as glucose, FFA and lactate during the haematopoietic stress response. Furthermore, amino acids are involved in the regulation of the signalling pathways that determine the metabolic profile of HSCs. Branched chain amino acids have been shown to regulate levels of MEIS1 and p21, which are both involved in the maintenance of HSC quiescence and expansion (49). Amino acids, particularly glutamine have also been shown to play a role in HSC differentiation (33). Due to these many and somewhat contrasting effects, the roles of amino acids in HSC metabolism cannot at present be simply defined. It is likely amino acid function in HSC is dependent of the broader cellular and niche context. In AML, there is increasing evidence that the malignant cells utilise amino acids overall for growth advantage (16) and it seems likely that as with other pathways the tumour hijacks mechanisms inherent to HSCs in order to maximise growth potential. Thus, the increased understanding of amino acid metabolism in AML may help us to improve our knowledge of their role in HSCs both in the steady state and in response to stress.

## How AML Manipulates the BM Niche to Drive its Metabolic Requirements

### Mitochondrial Transfer

**Figure 2:**
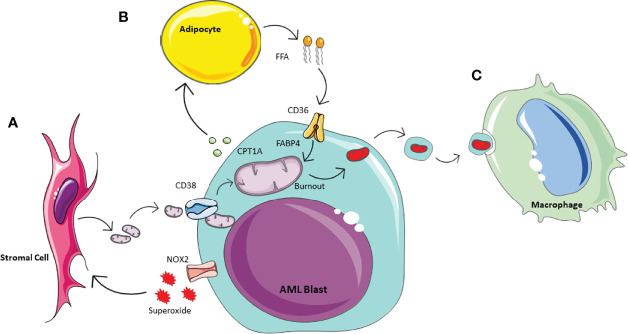
Schematic of AML metabolic of bone marrow microenvironment. Acute Myeliod Leukaemia (AML) is able to manipulate the surrounding environment to enhance its own metabolism. **(A)** In response to the increase energy demanding, AML produces NOX2 derived superoxide, whichacts on BM stormal cells to activate mitochondrial transfer. Mitochondrial uptake by AML is enable through tunneleing nanotubules by the transmembrane glycoprotien CD38. **(B)** AML manipulates adipocytes inducing phosphorylation of lipase, to activate lipolysis and te release of Free Fatty Acids (FFA). AML is able to icrease FFa uptake via increased expression of CD38 and fatty-acid binding protiens (FABPs). **(C)** To prevent the activation of apoptotic pathways, AML outsources its waste mitochondria. This process of outsourcing mitophagy utilises the macrophages in the microenvionment.

Historically is was thought that cancer cells preferentially generate ATP through anaerobic glycolysis, as described by Warburg in 1956 (50). However, it has since become apparent that the metabolic processes of malignant cells are much more complex, and it has been suggested AML in fact has a bias towards mitochondrial OXPHOS (51). AML blasts have a very high proliferative potential and therefore equally high energy requirements. Thus it is not surprising that they utilise this most efficient form of ATP production, however as a result they have a need to increase their mitochondrial mass (52). It has been shown that they achieve this by hijacking the HSC response to stress to acquire mitochondria from BM stromal cells in their leukaemic niche (10, 17, 53). Studies have shown this can occur *via* multiple mechanisms in AML including transfer in extracellular vesicles or *via* leukaemia-derived tunnelling nanotubes, which requires cell-cell interactions (13, 17, 54, 55). As with HSCs and progenitor cells, increased ROS levels induce mitochondrial transfer. AML blasts can create oxidative stress in the BM stromal cells *via* NADPH oxidase-2, which generate superoxide (13) (**Figure 2A**). These conditions create a pro-tumoural environment in which AML blasts can acquire the mitochondria needed to generate the energy required for their increased proliferation. Understanding how AML blasts can maintain their high energy production required for their survival and proliferation can help to inform future treatment strategies. The transfer of mitochondria from BM stromal cells to AML blasts has been shown to rely on the transmembrane glycoprotein CD38 (56). This can be inhibited with the monoclonal antibody targetting CD38, already used widely in the treatment of myeloma (57) and preclinical data suggest it may be effective in treating AML (58, 59). The anti-CD38 antibody Daratumumab for example, has been shown to alter the metabolic profile of AML blasts and inhibit disease progression *in vivo* (56). This exemplifies how insight into the metabolic changes of malignant cells may be utilised to identify potential targets and inform future treatment strategies.

During normal haematopoiesis, long-term HSC health and quiescence is dependent on maintaining low levels of ROS (60, 61). By changing this fundamental aspect of the BM niche, AML not only impairs healthy haematopoiesis but it also has to adapt itself so that is can thrive in this environment (62). In fact some mutations seen in AML including the Fms-like tyrosine kinase 3 (FLT-3) receptor mutations, which occurs in 30-35% of cases (63) and is associated with particularly poor prognosis, are known to enhance ROS production by AML blasts further (64, 65) and high ROS levels are known to drive leukaemogenesis (65, 66). This creates a particular challenge in the treatment of AML, as many of the traditional chemotherapy agents, including daunorubicin and cytarabine, are known to increase ROS levels in order to promote apoptosis (67). Whilst even AML cells cannot withstand unlimited levels of ROS and these treatments are therefore effective to an extent, the increased levels of ROS also contribute to genomic instability, which further favors AML survival and promotes chemotherapy resistance (68, 69). Whether ROS production and its downstream effects on AML progression could be targeted in combination with other existing treatments remains to be explored (62). It is clear however that manipulation of ROS levels in the BM niche and the adaptability of AML blasts to tolerate this far better than HSCs provides another survival advantage to AML.

### Mitochondrial Removal

As the AML blasts acquire increasing numbers of mitochondria from the environment, they also accumulate dysfunctional mitochondria and other waste products. If not removed, dysfunctional mitochondria will initiate intra-cellular apoptotic pathways. Therefore, to limit this and minimise cell death, dysfunctional mitochondria are release in extracellular vesicles by AML blasts (70). These are then phagocytosed and removed from the microenvironment by macrophages (**Figure 2C**). The concept of outsourcing mitophagy has also been shown in mesychymal stem cells where this is mediated by arrestin domain-containing protein 1 (71). Thus, AML blasts are able to utilise another cell in their environment to help with the removal of ‘waste products’ which could otherwise be detrimental to their growth.

### Amino Acid Metabolism

Although the AML blasts can acquire more mitochondria from their environment, they also need to maintain a constant supply of the substrates for OXPHOS and utilise as many metabolic pathways as possible to keep up with the energy supply demanded from rapid proliferation. As well as directly contributing to energy production substrates including glucose, lactate, amino acids and FFAs all feed into the TCA cycle to maximise energy output. As already described by Warburg, malignant cells including AML cells can increase their glucose uptake and upregulate their glucose metabolism (72, 73). Whilst we now know this is not solely to directly generate ATP through glycolysis but also to provide sufficient pyruvate for the TCA cycle and subsequent glycolysis, it nevertheless is an important component of the leukaemic metabolic profile. Another by-product of glycolysis is lactate which is known to promote a pro-tumoral microenvironment and has therefore been identified as a potential treatment target (74, 75). However, it is also evident that the conversion of pyruvate to lactate is reversible and therefore lactate can be converted back to pyruvate to then feed into the TCA cycle.

Amino acid metabolism presents a further source of fuel for ATP production in cancer cells (76–79). Cancer cells increase their uptake of amino acids which have a number of functions including protein biosynthesis, activation of signalling pathways as well as a substrate for the TCA cycle (80). Metabolism of amino acids, particularly glutamine, glutamate, and proline, has been shown to be increased in leukaemic stem cells isolated from AML patients (16). Glutamine is the most abundant amino acid in the human body and not only provides metabolites for the TCA cycle but is also required for numerous essential cell functions, including the production of antioxidants to regulate ROS, cell signalling and the synthesis of other amino acids, lipids and nucleic acids (81). Glutamine is a nitrogen donor and provides an amide group for *de novo* nucleotides and therefore can be a rate limiting factor for AML cell proliferation (81). The oncogenic protein c-Myc has a regulatory function on cell metabolism and can shift mitochondrial metabolism to increase its reliance on glutamine, promoting glutaminolysis, as well as increasing demethylation and inducing glutamine synthetase expression (82). The reliance of leukaemic stem cells on amino acid metabolism for production of ATP and the building of proteins for proliferation, makes them more susceptible to treatments targeting amino acid pathways (16). The combination treatment of venetoclax, a BCL-2 inhibitor and azacytidine, a hypomethylating agent, is now commonly used to treat older patients who are unable to tolerate more intensive treatments. Analysis has shown that treatment with venetoclax and azacytidine disrupts the TCA and OXPHOS in leukaemic stem cells (83) and that this results from a reduction in amino acid uptake (16). Thus, highlighting the potential therapeutic benefits of targeting the energy supply and metabolism of the leukaemic blast.

### Adipocytes and FFA Metabolism

Similarly to HSCs in stressed conditions, AML blasts utilise β-oxidation of FFAs by accessing the abundant BM adipose tissue to further increase their energy supply to maintain their continuous and rapid self-renewal and differentiation capacity (11) (**Figure 2B**). It has been shown that a subset of leukaemic stem cells have upregulated CD36 expression to promote the use of FFA as a source of energy, and reside in an adipocytic niche (15). The proximity to adipocytes also increases the expression of fatty acid binding protein-4 (FABP4), another protein that aids the transfer of FFA, into AML blasts (11, 84). Furthermore, AML blasts can have a direct impact on adipocyte metabolism and induce phosphorylation of lipase to activate lipolysis and release FFA (84). This increase in FFA release combined with the increased expression of CD36 and FABP4 improves uptake of FFAs by AML blasts providing the necessary fuel for β-oxidation and therefore increased OXPHOS (84). The role of adipose tissue in AML survival and potential treatment resistance is of particular interest as the BM composition changes with age with an increase in BM adipose tissue (85) and AML is primarily a disease of the elderly (86). FFA metabolism has shown to play a particular role in relapsed AML, where they compensate for the reduced amino acid metabolism that is observed after some treatments which inhibit these pathways (16). Thus, the interaction between AML blasts and adipose tissue may be a useful target in the treatment of AML especially in the older population and in the prevention and treatment of relapsed disease.

## Conclusion

AML is a highly proliferative malignancy, which for many is associated with poor clinical outcomes, even despite intensive chemotherapy regimens. While Warburg stated that all cancers preferentially undergo glycolysis for production of ATP (50), in recent years it has become evident that the metabolic profile of AML blasts is more complex and there is a bias towards utilising the most abundant metabolites in the BM niche (11, 13, 16). With this in mind it is therefore predictable that AML blasts would modulate the BM niche to create a pro-tumoral environment that supports their own growth over normal haematopoiesis. AML blasts utilise the pathways intended for the effective metabolic response of HSCs to stress in order to acquire the energy required for their survival, chemotherapy evasion and relentless high levels of proliferation (5, 87). Some of these pathways are already targeted by existing treatments, however improved understanding of these mechanisms will help to elucidate new targets and better tolerated treatments for this disease.

## Author Contributions

RM, CH, KB and SR conceptualised and wrote the paper. All authors contributed to the article and approved the submitted version.

## Funding

CH is funded by Wellcome Trust Clinical Research Fellowship (220534/Z/20/Z) and was supported by the NNUH charitable fund. The work was supported from the MRC project grant SAR (MR/T02934X/1).

## Conflict of Interest

The authors declare that the research was conducted in the absence of any commercial or financial relationships that could be construed as a potential conflict of interest.

## Publisher’s Note

All claims expressed in this article are solely those of the authors and do not necessarily represent those of their affiliated organizations, or those of the publisher, the editors and the reviewers. Any product that may be evaluated in this article, or claim that may be made by its manufacturer, is not guaranteed or endorsed by the publisher.
